# Effectiveness of Smartphone-Based Cognitive Behavioral Therapy Among Patients With Major Depression: Systematic Review of Health Implications

**DOI:** 10.2196/24703

**Published:** 2021-02-10

**Authors:** Robert Hrynyschyn, Christoph Dockweiler

**Affiliations:** 1 Charité – Universitätsmedizin Berlin, corporate member of Freie Universität Berlin, Humboldt-Universität zu Berlin, and Berlin Institute of Health, Institute of Health and Nursing Science Berlin Germany; 2 Centre for ePublic Health Research School of Public Health Bielefeld University Bielefeld Germany

**Keywords:** mobile health, depression, cognitive behavioral therapy, systematic review, mobile phone

## Abstract

**Background:**

Depression is often associated with rapid changes in mood and quality of life that persist for a period of 2 weeks. Despite medical innovations, there are problems in the provision of care. Long waiting times for treatment and high recurrence rates of depression cause enormous costs for health care systems. At the same time, comprehensive limitations in physical, psychological, and social dimensions are observed for patients with depression, which significantly reduce their quality of life. In addition to patient-specific limitations, undersupply and inappropriate health care have been determined. For this reason, new forms of care are discussed. Smartphone-based therapy is considered to have great potential due to its reach and easy accessibility. Low socioeconomic groups, which are always difficult to reach for public health interventions, can now be accessed due to the high dispersion of smartphones. There is still little information about the impact and mechanisms of smartphone-based therapy on depression. In a systematic literature review, the health implications of smartphone-based therapy were presented in comparison with standard care.

**Objective:**

The objective of this review was to identify and summarize the existing evidence regarding smartphone-based cognitive behavioral therapy for patients with depression and to present the health implications of smartphone-based cognitive behavioral therapy of considered endpoints.

**Methods:**

A systematic literature review was conducted to identify relevant studies by means of inclusion and exclusion criteria. For this purpose, the PubMed and Psyndex databases were systematically searched using a search syntax. The endpoints of depressive symptoms, depression-related anxiety, self-efficacy or self-esteem, and quality of life were analyzed. Identified studies were evaluated for study quality and risk of bias. After applying the inclusion and exclusion criteria, 8 studies were identified.

**Results:**

The studies examined in this review reported contradictory results regarding the investigated endpoints. In addition, due to clinical and methodological heterogeneity, it was difficult to derive evident results. All included studies reported effects on depressive symptoms. The other investigated endpoints were only reported by isolated studies. Only 50% (4/8) of the studies reported effects on depression-related anxiety, self-efficacy or self-esteem, and quality of life.

**Conclusions:**

No clear implications of smartphone-based cognitive behavioral therapy could be established. Evidence for the treatment of depression using smartphone-based cognitive behavioral therapy is limited. Additional research projects are needed to demonstrate the effects of smartphone-based cognitive behavioral therapy in the context of evidence-based medicine and to enable its translation into standard care. Participatory technology development might help to address current problems in mobile health intervention studies.

## Introduction

### Background

Depression can affect mental and physical health. It is estimated that nearly 322 million people worldwide have depression, and the World Health Organization (WHO) has stated that depression is the single largest factor contributing to global disability [1]. Recently, findings from the Global Burden of Disease study stated that the incidence of depression has increased by approximately 50% in 2017 compared with that in 1990 [2]. Depression is equally prevalent in high-income countries and middle- to low-income countries [3]. In addition, the European Health Interview showed that the European average accounts for approximately 6.6% of people with depression [4]. Due to the widespread prevalence of depression and its rising burden, the social and health policy significance of mental illnesses is increasing [5]. Therefore, depression is a major global public health domain of the 21st century, and its importance is increasing.

Depression is associated with changes in mood and quality of life that persist for a period of 2 weeks [6]. Furthermore, a decline in activity, loss of appetite, or prolonged fatigue is observable. In addition, depression is associated with higher mortality rates, which can be explained by depression-related suicide and an unhealthy lifestyle that can cause diabetes or cardiovascular diseases. Suicide is a major cause of injury and death worldwide [7]. Approximately 15% of patients with depression face suicidal thoughts [8].

In addition to the rising numbers and the increasing burden of disease, mental illnesses have major economic influences. The Federal Statistical Office of Germany reported that in 2019, direct costs of approximately 8.7 billion Euros (US $ 10.5 billion) were caused by depression in Germany. It was estimated that 4.6 billion Euros (US $ 5.5 billion) were spent on the inpatient sector and 3.3 billion Euros (US $ 4.0 billion) were spent on the outpatient sector. This corresponds to approximately 2.6% of the total costs in the health care system [9]. In addition, approximately 42% of early retirements were found to be associated with mental disorders. A health report published by the German insurance company Deutsche Angestellten Krankenkasse ranked mental disorders third in 2019, causing 15.2% of all sick leave days in Germany annually. Only respiratory and musculoskeletal disorders have caused more sick leave days than mental disorders [10,11].

In the past, people with a diagnosis of mental illness faced social consequences such as marginalization, stigmatization, and isolation [12]. However, in recent years, public awareness has increased, and patients with depression should no longer feel the need to conceal their diagnosis. A correct diagnostic classification by the general practitioner and early assessment of treatment needs can significantly contribute to less concealment of diagnoses and public awareness [13].

Even today, people with depression face different barriers to health care access. In contrast to chronic diseases with no reversibility, depression is a treatable disease. Only 25% of depressed patients receive accurate care, and in Germany, approximately 50% are not provided with medical guidelines-oriented care [14]. There are both patient-level and system-level reasons for this undersupply and existing barriers. Patient-level barriers are associated with time, transportation constraints, or a considerable cost. System-level barriers include long waiting lists and a dearth of therapists and doctors, especially in rural areas and low-income countries [15,16]. High-income countries can provide, on average, 72 mental health workers per 100,000 individuals, whereas low-income countries can only provide approximately 2 mental health workers per 100,000 individuals [7].

To overcome these barriers, we need innovative ways to reduce waiting times and increase professional treatment by mental health workers. Due to the increasing use of information and communication technologies, mobile health (mHealth) is considered to have great potential in the health care sector. Especially for rural care concepts, mHealth is a promising tool to improve care in structurally weak areas. Here, the cross-sectoral and interprofessional approach is particularly suitable, as it can lead to a reduction in the care interruptions between outpatient and inpatient sectors [17].

Comparison of e-mental health treatments with traditional face-to-face treatments suggests that technology is not predominantly accepted by patients, although it may still provide solutions to address the patient-level and system-level barriers [18]. Access issues can easily be mitigated using technological innovations. Several studies have demonstrated that telemedicine can be used for remote treatments [19,20]. First-wave technology-based movements such as internet-based cognitive behavioral therapy (CBT) proved their potential in different meta-analyses, which demonstrated both their safety and effectiveness [21,22].

The promising results of treating patients through distance have resulted in considerable interest in transferring internet-based and computerized techniques to smartphone apps. The crucial factor of smartphone-based apps can be seen in their tremendous reach. Patients can also receive therapy whenever they need it most without taking an appointment with their therapist, which often results in long waiting times with possible aggravation of symptoms [23]. Currently, thousands of mental health apps are available in the market, and these apps are designed for a brief frequent use throughout the day [24]. Considering how fast mental health apps are developed and implemented, it is important to address the challenges that these developments yield. We will face 3 major challenges in the future: (1) issues of low engagement with digital mental health tools, (2) lack of sufficient evidence, and (3) poor understanding related to security issues among target groups [25].

There is a lack of evidence for efficacy of smartphone-based interventions, which is essential for integrating new provisions into future health care. Although hundreds of mental health apps are available on unregulated app stores, there are only a few proof-of-concept studies and small-scale randomized controlled trials (RCTs) that evaluate the effectiveness of smartphone-based CBTs for depression [26]. Consequently, it is critical to ensure that patients and clinicians have enough information to understand evidence-based digital treatments for depression. Recent meta-analyses have documented positive effects on diabetes [27] and anxiety [28]; however, these effects still need to be measured for depression.

### Objectives

Therefore, the aim of this study was to close this existing research desideratum. A systematic review was conducted to examine the effects of smartphone-based CBTs for the treatment of depression. Therefore, the leading research question was as follows: Are smartphone-based CBTs effective for treating depression and do they improve depression-related clinical endpoints?

## Methods

### Search Strategy

The used literature was determined by a systematic search. A systematic search was conducted in the PubMed and Psyndex databases. Relevant articles on smartphone-based therapy and depression were collected and evaluated. The searched terms were extended by relevant keywords of the articles found and supplemented by Medical Subject Headings (MeSH). Three generic terms were identified for which related terms were collected in English (Table 1). To obtain more results, the words were connected using the Boolean operators *AND* and *OR*. If the identified articles appeared to be relevant, the summaries and available full texts were read. Further articles were found by viewing the source references in the articles read.

**Table 1:** Search matrix.

Topic	Search terms
Depression	Depression (MeSH^a^); depress; major depression; depressive disorder (MeSH); depressive disorder, major (MeSH); depressive episode; unipolar depression
Cognitive behavioral therapy	Cognitive therapy; behavior therapy (MeSH); cognitive behavioral therapy (MeSH); acceptance and commitment therapy (MeSH), mindfulness
Smartphone-based interventions	Smartphone (MeSH); computers, handheld (MeSH); mHealth; mobile health; smartphone-delivered therapy; smartphone-based therapy; internet-based interventions (MeSH); mobile applications (MeSH)

^a^MeSH: Medical Subject Headings.

### The Population, Intervention, Comparison, Outcome, Study Search Strategy

Inclusion and exclusion criteria were developed a priori for the systematic review. For this purpose, the PICOS (population, intervention, comparison, outcome, study) search strategy was used in the context of evidence-based medicine [26,29]. The search strategy was based on population, intervention, comparison, outcome, and study type.

### Population

Studies were included if they dealt with people with mild to moderate depression or depressive symptoms. Furthermore, the study participants needed to be aged at least 18 years.

Studies were excluded if they dealt with subpopulations such as children or minority groups (refugees or only specific ethnic communities). Furthermore, studies were excluded when they assessed depression as a comorbidity of other relevant diseases (eg, chronic backpain, diabetes, and cancer). In addition, studies with treatment of other forms of depression such as postpartum depression, severe courses of depression with psychotic events, and schizophrenia were excluded.

### Intervention

Studies that focused solely on the provision of CBT via smartphones or tablets or an additional treatment via smartphones or tablets in combination with treatment as usual were included. It was mandatory that the therapy provision be delivered by smartphone or tablet apps.

On the contrary, studies were excluded if they used other therapy delivery formats than smartphone or tablet apps. Studies that merely provided telephone support were excluded. As the aim of this study was to determine the effectiveness of smartphone-delivered therapy formats, computerized therapy formats were excluded. Web-based apps with no smartphone or tablet involvement were also excluded.

### Comparison

To include studies in the systematic review, a control group (CG) needed to be present. The CG can be treated as usual, an active control comparison (eg, other treatment apps), or inactive control comparison. Studies that did not provide a CG were excluded.

### Outcomes

This systematic review assessed clinical outcome points, which are associated with depression. The following clinical outcomes were assessed: depressive symptoms, depression-related anxiety, self-efficacy or self-esteem, and quality of life. Studies with the abovementioned outcomes as primary or secondary outcomes were included. If the study investigated one of the mentioned endpoints, it was included in the review. Studies that did not consider the investigated endpoints or did not report results were excluded.

### Study Design

To provide the highest degree of evidence, RCTs were considered relevant to analyze the stated hypothesis.

Studies that that had other study designs or lacked a CG were excluded even if they were described as an RCT. Furthermore, owing to the large number of studies conducted in this field, the time interval was limited to consider the most relevant studies. Studies conducted before 2015, indicating a time interval of the last 5 years, were excluded. Studies published in German or English were included.

After a search syntax was developed using the search matrix and the appropriate MeSH terms as part of the systematic literature search, articles were identified in PubMed and Psyndex (Multimedia Appendix 1). The search was last updated in May 2020. The inclusion and exclusion criteria were applied in a 2-stage process. First, the titles and abstracts of the studies were reviewed using the inclusion and exclusion criteria. Duplicates and studies that did not meet the inclusion criteria were excluded. If the studies appeared adequate or an assessment based on the title and abstract was not possible, the full texts were read.

In the second step, the inclusion and exclusion criteria were applied again to the full texts. The full texts that did not meet the inclusion criteria were excluded. In addition to the systematic database search, a hand search was carried out in which further relevant studies were identified according to the inclusion and exclusion criteria. The studies identified by hand search also underwent a 2-stage process.

### Quality Assessment

The publications included in the qualitative synthesis were assessed for quality. The instrument described by Hailey et al [30] was used for the quality assessment, which was extended by the method described by Polisena et al [31]. Their approach to the appraisal of study quality relies on the summation of 2 scales dealing with study designs and study performance (Textbox 1 [31]). The authors state that both scales are ordinal, as for each of the 5 study performance attributes, it is hypothesized that they have the same impact on the quality of the study. The scale for study design is also ordinal and values the increasing confidence in the different designs and their relevance for decision making.

Hailey et al [30] distinguished 4 different study designs for quality assessment, which were assigned different scores. RCTs with at least 50 participants per study arm received the highest score. Retrospective, nonrandomized studies received the least number of points. Polisena et al [31] further differentiated the RCTs. Here, half a point was deducted if the randomization was not described correctly or if blinding was not performed or described.

**Instrument to assess the quality of the included studies.**

Study category:

- Study design
  Big randomized controlled trial (RCT; ≥50 participants each intervention arm): 5 points
  Small RCT (<50 participants each intervention arm): 3 points
  Prospective nonrandomized trial: 2 points
  Retrospective nonrandomized trial: 1 point
- If RCT (half a point is deducted if the information is missing)
  Description of randomization
  Implementation of blinding
  Description of blinding
- Study performance (0=no information, 1=information limited, 2=information satisfactory)
  Patient selection
  Description of the intervention
  Specification and analysis of study (intention-to-treat)
  Patient disposal
  Outcomes reported
- Resulting quality categories
  11.5-15.0 points: high quality (A)
  9.5-11.0 points: good quality (B)
  7.5-9.0 points: fair to good quality (C)
  5.5-7.0 points: poor to fair quality (D)
  1.0-5.0 points: poor quality (E)

A total of 5 study performance attributes were evaluated in the studies. Patient selection, description of the intervention, specification of the analysis, patient dropouts, and reported outcomes were considered. Two points were given when the information was sufficiently presented. A lack of information was rated with zero points. This system allowed to build a score to categorize the studies in terms of quality. The maximum score was 15 points for a study. Studies that achieved a minimum score of 11.5 points were classified in category A, which is the highest quality category. Scores between 9.5 and 11 points were of good quality and corresponded to category B. Quality categories of classes C to E (score below 9) showed fair to poor quality. These studies have considerable limitations that need to be considered when interpreting the results [30].

### Risk of Bias Assessment

Although the terms bias and quality are often used as synonymous concepts to evaluate included studies in the systematic reviews, the Cochrane Handbook distinguishes between those terms for the following reasons. First, the key aim of a review is to consider the extent to which the results of the included studies should be considered. Second, a study may have been performed with the highest possible standards but still have an important risk of bias. Third, the risk of bias assessment can be instrumental in overcoming the ambiguity between quality of reporting and the quality of underlying research. Finally, some markers of quality in medical research (eg, ethical approval or performing sample size calculations) are unlikely to have implications for the risk of bias [32]. Therefore, it is necessary to additionally perform a risk of bias assessment, although the quality assessment and the risk of bias assessment might have overlapping issues (eg, evaluation of randomization).

In this systematic review, the risk of bias assessment was used to evaluate the sequence generation, allocation concealment, blinding of participants and personnel, blinding of outcome assessment, and assessment of outcome data. The Cochrane Handbook for Systematic Reviews of Intervention (Version 5.1.0) was used as a basis for decision making, and the risk of bias assessment was conducted using Review Manager 5.3. (Cochrane Collaboration). By systematically screening the included studies for different biases, either low risk, unclear risk, or high risk evaluations were made.

## Results

### Overview

**Figure 1:**
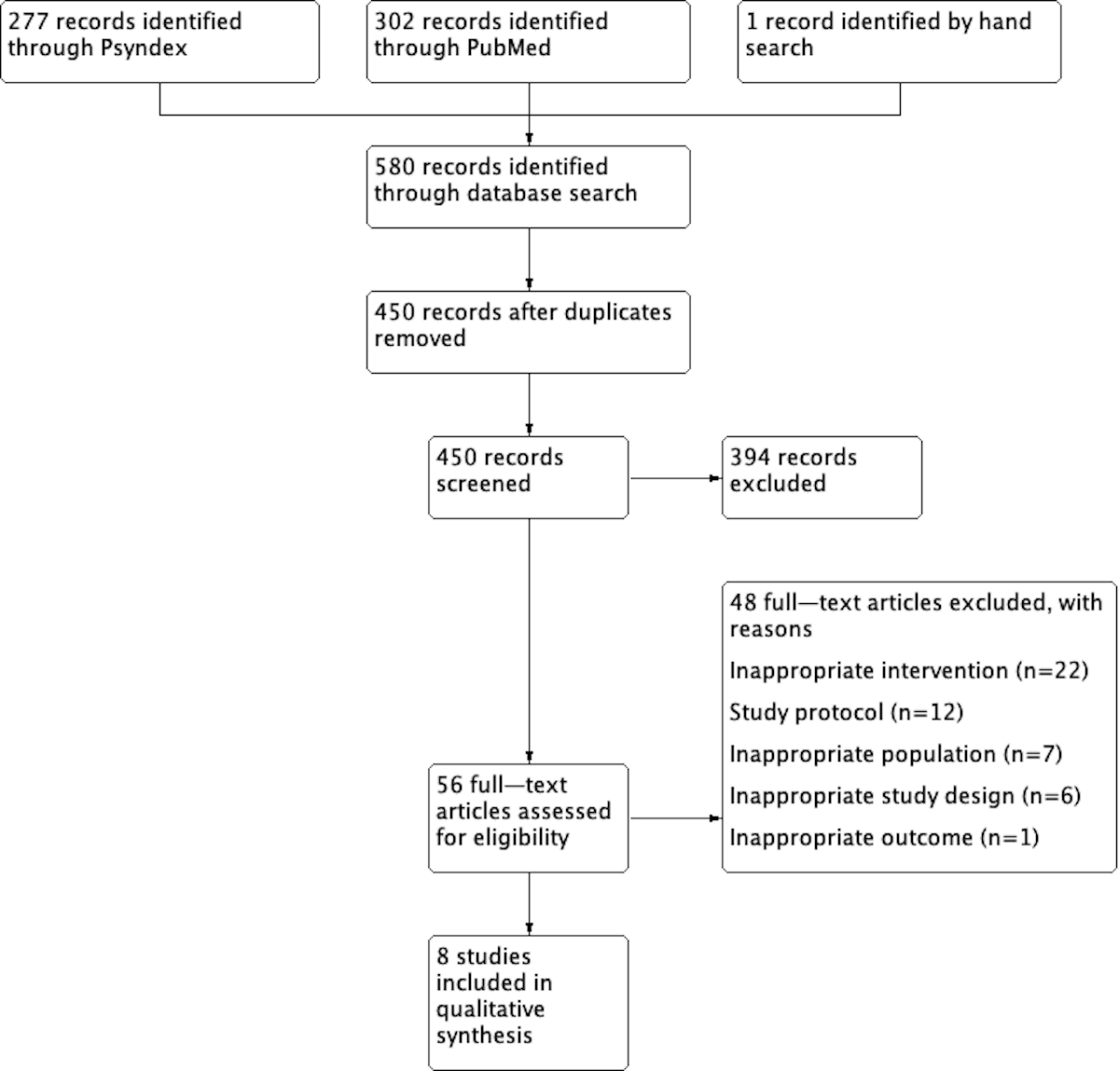
Process of study selection.

A total of 8 studies were included in this systematic review. The process of study selection is further described in Figure 1. After the search syntax was developed and entered in PubMed and Psyndex, 580 studies were identified. A total of 302 records were identified via PubMed, and 277 records were found in Psyndex. One additional record was identified by hand search, systematically reviewing the references of other studies. After duplicates were removed, 450 records were eligible for the first screening. The first screening was done by checking the abstract and title of the studies and evaluating them for suitability. A total of 394 records were excluded, resulting in 56 studies that were included for full-text assessment. From these 56 studies, 48 studies were excluded for different reasons. The main reason for exclusion of studies was an inappropriate intervention (22/48, 46%). This was followed by study protocols (12/48, 25%), an inappropriate population (7/48, 15%), and inappropriate study design (6/48, 13%). One study reported inappropriate outcomes.

The following sections will descriptively present and compare study and patient characteristics at baseline. Subsequently, the studies were analyzed regarding the considered endpoints, namely, depressive symptoms, depression-related anxiety, self-efficacy or self-esteem, and quality of life. The results of the systematic review are presented in Table 2. Multimedia Appendix 2 [16,33-39] presents an overview of the patient characteristics. The results of the regarded endpoints are shown in Multimedia Appendix 3 [16,33-39]. The patient characteristics are presented in Multimedia Appendix 2.

**Table 2:** Study characteristics.

Author, year, reference	Country	Evaluation period^a^	Financing	Participants	Intervention	Control	Outcomes	Instruments
Roepke et al, 2015 [35]	United States	6 weeks	Private	283 participants (93 control, 190 intervention) who owned an iPhone and had a CES-D^b^ score >16, aged >18 years	Intervention 1: Superbetter CBT^c^ app; Intervention 2: general Superbetter self-esteem and acceptance app	Waitlist	Depression Anxiety Life satisfaction Self-efficacy Social support Technology use Treatment strategies Daily functioning	CES-D GAD-7^d^ SWLS^e^ NGSE^f^ MSPSS^g^
Ly et al, 2015 [37]	Sweden	24 weeks	State	93 participants (47 control, 46 intervention) who owned a smartphone and had a PHQ^h^ score of ≥5, aged >18 years	4 face-to-face sessions and apps between the sessions, with behavioral activation components	10 face-to-face behavioral activation sessions	Depression Anxiety Quality of life Psychological flexibility	BDI-II^i^ PHQ-9 BAI^j^ QOLI^k^ AAQ-II^l^
Arean et al, 2016 [16]	United States	12 weeks	State	626 participants (206 control, 420 intervention) who owned a smartphone and had a PHQ-9 score of >5, aged >18 years	Intervention 1: cognitive training app (EVO); Intervention 2: evidence-based psychotherapy app (iPST)	Health tips	Depression Disability	PHQ-9 SDS^m^
Bakker et al, 2018 [39]	Australia	4 weeks	Not specified	312 participants (78 control, 234 intervention)	Intervention 1: MoodPrism self-monitoring mood tracking app; Intervention 2: MoodMission app for recommendation of CBT strategies; intervention 3: MoodKit CBT-based app	Waitlist	Depression Anxiety Well-being Emotional self-awareness Coping self-efficacy Mental health literacy	PHQ-9 GAD-7 WEMWBS^n^ ESAS-R^o^ CSES^p^ MHLQ^q^
Hur et al, 2018 [38]	South Korea	3 weeks	State	48 participants (24 control, 24 intervention)	Todac scenario-based mobile app	Mood charting app	Dysfunctionality Depression Anxiety Self-esteem Quality of life	DAS^r^ BDI-II STAI-X2^s^ RSES^t^ QOL^u^
Lüdtke et al, 2018 [36]	Germany	4 weeks	Not specified	90 participants (45 control, 45 intervention) who owned an iPhone, aged >18 years	Be Good to yourself self-help CBT app	Wailtlist	Depression Self-Esteem Quality of life Willingness to change Client satisfaction	PHQ-9 RSES WHOQOL^v^ URICA^w^ CSQ-8^x^
Dahne et al, 2019 [33]	United States	8 weeks	State	52 participants (28 control, 24 intervention) who owned a smartphone and had a PHQ-8 score of >10	Moodivate self-help behavioral activation app	Control 1: MoodKit CBT app; control 2: TAU^y^	Depression Feasibility	PHQ-8 BDI-II
Stiles-Shields et al, 2019 [34]	United States	10 weeks	State	30 participants (10 control, 20 intervention)	Intervention 1: Boost Me behavioral activation app Intervention 2: Thought Challenger cognitive therapy app	Waitlist	Depression App usability	PHQ-9 SUS^z^

^a^Follow-Up Assessment included.

^b^CES-D: Center for Epidemiologic Studies Depression Scale*.*

^c^CBT: cognitive behavioral therapy*.*

^d^GAD: Generalized Anxiety Disorder*.*

^e^SWLS: Satisfaction with Life Scale*.*

^f^NGSE: New General Self-Efficacy Scale*.*

^g^MSPSS: Multidimensional Scale of Perceived Social Support*.*

^h^PHQ: Patient Health Questionnaire*.*

^i^BDI: Beck Depression Inventory*.*

^j^BAI: Beck Anxiety Inventory*.*

^k^QOLI: Quality of Life Inventory*.*

^l^AAQ-II: Acceptance and Action Questionnaire*.*

^m^SDS: Sheehan Disability Scale*.*

^n^WEMWBS: Warwick-Edinburgh Mental Well-being Scale*.*

^o^ESAS-R: Emotional Self-Awareness Scale*-*Revised*.*

^p^CSES: Coping Self-Efficacy Scale*.*

^q^MHLQ: Mental Health Literacy Questionnaire*.*

^r^DAS: Dysfunctional Attitude Scale*.*

^s^STAI: Stat-Trait Anxiety Inventory*.*

^t^RSES: Rosenberg Self-Esteem Scale*.*

^u^QOL: Quality of Life*.*

^v^WHOQOL: World Health Quality of Life*.*

^w^URICA: University of Rhode Island Change Assessment Scale*.*

^x^CSQ: Client Satisfaction Questionnaire*.*

^y^TAU: treatment as usual*.*

^z^SUS: System Usability Scale*.*

### Description of Study Characteristics

The selected studies were compared in terms of the country of intervention, number of cases, investigation period, and source of funding. Interventions were also presented according to the endpoints, their applied instruments, and the technology used. Finally, the quality of the studies was assessed.

The implementation of the included studies was conducted in different regions of the world. Most of the studies (4/8, 50%) have been conducted in North America. All studies were performed in the United States [16,33-35]. Two studies have been conducted in Northern Europe, with 1 study completed in Germany [36] and 1 in Sweden [34,37]. Two studies evaluated the effectiveness of smartphone-based therapy in South Korea [38] and Australia [39].

The majority of studies (5/8, 63%) reported state funding. Of the 4 identified studies conducted in the United States, 3 were financed by the National Institute of Mental Health [16,33,34]. Two studies did not provide information on research funding [36,39], and 1 study was funded by private donations [35].

The evaluation periods ranged from 3 weeks in the study by Hur et al [38] to 24 weeks in the study by Ly et al [37]. In these evaluation periods, the follow-up assessment was included. Three studies had a shorter or equal evaluation period of 1 month [36,38,39], and in 4 studies, the period was shorter than 3 months [16,33-35].

In total, 1534 patients were evaluated by the 8 included studies. The range of participant numbers was considerably different and varied from 30 to 626 (mean 341, SD 206.2; median 432), when the intervention group (IG) and CG were considered. All studies differentiated between a CG and IG. The number of IGs and CGs differed between the studies. Three studies compared an IG with a CG [36-38]. In addition, 4 studies had more than 1 IG [16,34,35,39], and 1 study was designed with 2 CGs [33]. The number of participants in the IG varied from 20 to 420 (mean 223, SD 145; median 268). Furthermore, the number of participants in the CG was considerably lower, ranging from 10 to 206 (mean 118, SD 62.8; median 164).

Although all studies measured depression-related symptoms as outcomes, the instruments to assess the symptoms were different. Patient Health Questionnaire (PHQ-9) and Beck Depression Inventory-II (BDI-II) were the most common instruments. Five studies used the PHQ-9 to assess depression-related symptoms [16,34,36,37,39]. Only Dahne et al [33] used the PHQ-8 to assess symptoms. The reason for using the eighth version of the PHQ instead of the ninth was not further explained by the authors. Three studies measured depression symptoms using the BDI-II [33,37,38], and 1 study evaluated the symptoms by using the Center for Epidemiologic Studies Depression Scale [35].

Depression-related anxiety was measured in 4 studies [16,35,37,38]. Two studies used the Generalized Anxiety Disorder (7-item) Scale [35,39], and 1 study used the State-Trait Anxiety Inventory-X2 [38] and the Beck Anxiety Inventory tools [37].

Self-esteem or self-efficacy was measured differently by 4 studies. Two studies used the Rosenberg Self-Esteem Scale (RSES) to assess self-esteem [36,38]. One study evaluated this outcome using the New General Self-Efficacy Scale [35] and the Coping Self-Efficacy Scale [39]. Both scales had good Cronbach α values between .89 and .96.

Finally, quality of life was only measured in 2 studies. Lüdtke et al [36] used the WHO Quality of Life survey (WHOQOL-BREF), and Ly et al [37] used the Quality-of-Life Inventory. Other measures that were conducted by different studies were client satisfaction, app usability, daily functioning, and psychological flexibility.

The CG, with whom the IG was compared, was considerably different between the studies. Four studies provided a waitlist CG, which provided any form of treatment [34-36,39]. Other treatment strategies (eg, medication, psychotherapy, or coaching) were also allowed. Two studies used an inactive CG by providing health tips [13,16] or asking patients to complete a mood charting app [38]. Ly et al [37] compared IG with 10 face-to-face behavioral activation sessions, indicating an active CG. Patients were supported by therapists, and a treatment that resulted in giving homework and setting individualized aims was also done. Finally, the study by Dahne et al [33] was the only study with 2 CGs. One CG was inactive, providing treatment as usual, and the other CG was active, giving patients access to the CBT app *MoodKit*, which included thought checking, mood tracking, journaling, and activity scheduling.

The interventions performed varied between the studies. Four studies had more than 1 IG, comparing different smartphone apps with each other [16,34,35,39]. Often, a CBT app was compared with a different smartphone-based therapy approach. For example, Arean et al [16] compared the cognitive training app *EVO* with an evidence-based psychotherapy app *iPST*, which is a problem-based therapy approach managing mood in 7 steps. Similar approaches were conducted by Roepke et al [35] and Stiles-Shields et al [34], which compared either a CBT app with a self-esteem acceptance app or behavioral activation app strategies with cognitive restructuring app therapies. The study by Bakker et al [39] was the only study with 3 IGs. The authors compared the self-monitoring app *MoodPrism*; a CBT recommendation app *MoodKit*, which suggested different strategies depending on the reported moods and anxious feelings; and the CBT app *MoodKit*. In contrast, Ly et al [37] applied smartphone-based apps as an additional component to psychotherapy using apps between psychotherapy sessions. Hur et al [38] applied a scenario-based app approach combined with a 3-step quiz in which cognitive distortions were identified and resolved with decatastrophizing approaches, similar to cognitive restructuring. In contrast to other studies, Dahne et al [33] provided a self-help behavioral activation app to treat patients. This self-help approach, which should help patients in their daily routine by providing access to cognitive strategies such as mindfulness-based and social competence skill exercises, was also used by Lüdtke et al [36].

The quality of the identified studies was good to very good (mean 11.43 points, SD 2,16; median 12 points). A total of 5 studies (approximately 63%) were assessed with the highest quality and were categorized as A. One study (approximately 12%) was evaluated with category B, and 2 studies (approximately 25%) were labeled with C. No study could reach the maximum score of 15 points. This may be due to difficulties in blinding in mHealth studies. In contrast to pharmaceutical studies with placebo controls, blinding is difficult to guarantee in mHealth studies. All studies were RCTs. Of these studies, 3 had more than 50 participants in the IG [16,35,39]. The other 5 studies presented less than 50 participants in the IG [33,34,36-38]. Detailed measurements that were performed concerning the study design or study performance are presented in Multimedia Appendix 4.

**Figure 2:**
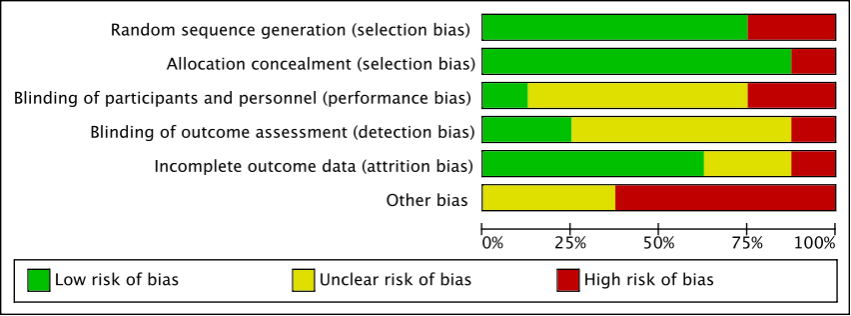
Risk of bias graph.

**Figure 3:**
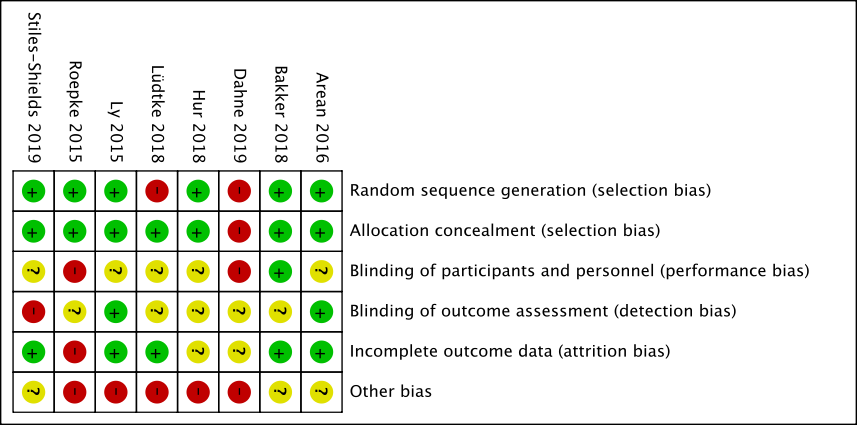
Risk of bias summary.

The performed risk of bias assessment of the included studies revealed different percentages concerning the analyzed biases. Low risks of bias have been found for random sequence generation and allocation concealment. In 75% (6/8) of the studies, a low risk of bias was determined. A total of 88% (7/8) of the studies performed proper allocation concealment, minimizing selection bias. The studies were inconsistent with regard to performance and detection bias. In 63% (5/8) of the studies, a low or high risk of bias could not be determined due to missing information. This was also evident for the detection bias, as the blinding of outcome assessment was often not described. The risk of attrition bias was relatively low in the studies (5/8, 63%). In contrast, the risk of other biases could not be ruled out, as sample sizes, gender biases, or intervention development for only 1 system software (either Android or iOS) could bias the reported effects. Therefore, the percentage of other biases was relatively high, accounting for 62.5% (5/8) of the studies that had a high risk of bias. Comparing the results of the quality assessment by Hailey et al [30] and the risk of bias assessment, a good agreement was found. The overall percentages are shown in Figure 2. Detailed bias characteristics of the included studies can be obtained from Figure 3.

### Description of Patient Characteristics

The overall mean age of the included patients was relatively similar. Only 1 study reported considerably lower ages in the IG and CG [38]. The authors reported the mean age to be 24.76 years (SD 3.70) in the IG and 22.65 years (SD 2.42) in the CG. The overall mean age of the patients in the IG ranged from 24.76 to 43.84 years (mean 35.99, SD 6.35; median 36.85) indicating that the studies observed a relatively young population. The patients in the CG were slightly younger than those in the IG. The lowest age was reported to be 22.65 years, whereas the highest age was reported to be 44.57 years (mean 35.36, SD 7.14; median 33.85). The study by Roepke et al [35] was the only study that reported conventional levels of statistical significance regarding age differences (*F*_2,280_=2.89; P=.06). The other studies either did not conduct statistical inference or did not report significant differences.

The included studies were highly heterogeneous regarding gender distribution. All studies reported a higher percentage of female patients participating than male patients. This was evident for IG and CG. The most balanced gender distribution for IG was found in the study by Ly et al [37]. They reported that 66% (30/46) were female patients and 35% (16/46) were male patients. The same study also reported the most balanced gender distribution for CG, indicating that 75% (35/47) were female and 26% (12/47) were male. In contrast, other studies had female participant percentages between 80% and 90% [34,36,39].

The observed patients in the included studies were moderately depressed with respect to baseline PHQ-9 values. Only 5 of the 8 studies reported baseline PHQ-9 values [16,34,36,37,39]. The PHQ-9 values ranged from 10.07 to 16.1 in the IG (mean 13.36, SD 2.52; median 13.63) and 8.55 to 16.1 in the CG (mean 13.27, SD 2.95; median 13.64). These mean PHQ-9 values indicate that the observed population had moderate depression [40].

Three studies assessed baseline depression symptoms using the BDI-II [33,37,38]. The results are comparable to the PHQ-9 values, indicating that the patients had moderate depression symptoms [41]. The BDI-II values ranged from 22.65 to 28.96 in the IG (mean 26.65, SD 3.48; median 28.35) and 25.59 to 32.33 in the CG (mean 28.41, SD 3.5; median 27.32). In contrast to the studies with PHQ-9 measurement, the CG of the studies with BDI-II reported higher values when compared with the IG.

The participants were recruited from the general population. Some studies also recruited patients from outpatient clinics [36,38,39]. Dahne et al [30] recruited patients only from outpatient clinics. Mostly, the recruitment strategies included advertisements on the internet (eg, Craigslist) or social media platforms (eg, Twitter). All studies excluded participants with suicidal thoughts or beliefs. Furthermore, some studies excluded participants who had different doses of antidepressants, comorbidities, alcohol problems, or were already treated by psychotherapy [34,37].

### Implications for Depression Symptoms

Data regarding depression symptoms were good. All studies investigated the effects on depression symptoms. However, the reported results of these studies are inconsistent and do not suggest clear implications for depression symptoms.

A total of 5 studies found that there was no significant difference between the IG and CG. Arean et al [16] determined a PHQ-9 score decrease of 0.73 points for the total sample. However, the models revealed no significant difference between the 2 IGs when compared with the CG (β-coefficient −0.01; P=.90). This was also evident in the mildly depressed subgroup analysis. Other subgroup analysis revealed that there was a significant difference for patients with a higher baseline depression at week 12 for the IG that received problem-solving therapy relative to the CG (t_201_=−2.36; P=.02). Dahne et al [30,33] showed that the between-group mean differences were not significantly different across time points. This was found for IG versus CG 2 (the notations are based on the names used in Table 2; mean difference −3.94; P=.18), IG versus CG 1 (mean difference 1.74; P=.55), and CG 1 versus CG 2 (mean difference −5.69; P=.07). The only significant between-group difference was observed in week 6, in which both IGs reported significantly lower depressive symptoms compared with the treatment as usual condition: IG versus CG 2 (mean difference −7.51; P=.02) and CG 1 versus CG 2 (mean difference −7.68; P=.03). The reported effect sizes were low, estimating Cohen *d* to be 0.33 for IG and 0.44 for CG 1. The study by Hur et al [38] was the only study that estimated the effects between the IG and CG using a nonparametric Wilcoxon rank-sum test. This test also revealed no significant differences between intervention and CG at follow-up (Z=−1.90; P=.06). Other studies conducted an analysis of variance to estimate the differences between intervention and control. Ly et al [37] determined an *F*_1,171.81_ value of 0.13 for BDI-II and *F*_1,911.85_=0.11 for PHQ-9, indicating that there was no significant difference between the IG and CG (P=.72 and .74, respectively). These results were supported by Lüdtke et al [36] who estimated an *F* value of 0.173 for PHQ-9 scores with a P value of .68.

Three studies estimated the positive effect of smartphone-based cognitive therapy for depression symptoms. Roepke et al [35] determined depression symptoms using the Center for Epidemiologic Studies Depression Scale and found that IG 1 significantly decreased symptoms relative to the waitlist CG (t_237_=−2.80; P<.01). In addition, a significant decrease was also found in IG 2, which applied a self-esteem and acceptance app (t_237_=−3.73; P<.001). When both IGs were compared, there was no significant difference between the groups (t_237_=0.82; P=.41), indicating that the CBT app was not superior to the self-esteem and acceptance app. The other 2 studies used the PHQ-9 to estimate depression symptoms. Both studies conducted an analysis of variance, which revealed significant differences. Bakker et al [39] compared 3 different interventions with the control condition. Only IG 2 and IG 3, which provided CBT strategies and CBT, showed significant decreases in depression symptoms. The decrease in symptoms was slightly higher in IG 3 (*F*=4.24; P<.05). The analysis of variance of IG 2 also revealed a significant *F* value of 4.39 (P<.05). In addition, the effect sizes were small partial eta–squared=0.035 (IG 3) and partial eta–squared=0.038 (IG 2). In contrast, IG 1 showed no significant effects relative to waitlist CG (*F*=0.78; P>.05). Stiles-Shields et al [34] estimated in a repeated-measures ANOVA (analysis of variance) a higher eta-squared effect size of 0.18 and a significant *F*_6_ value of 2.78 (P=.02), stating that the PHQ-9 scores differed significantly between group assignments. Post hoc analyses revealed that significant differences occurred between the IG 2 and the control condition (P=.03), but no significant differences were found between the behavioral activation app intervention and the other 2 groups (P>.2).

It was evident that the studies that compared a smartphone-based therapy app with a waitlist CG revealed significant differences. With the exception of the study by Lüdtke et al [36], all studies with a waitlist CG showed significant differences. Other studies that had active CGs that applied other smartphone-based therapy formats or support between face-to-face sessions could not establish the superiority of smartphone-based CBT [33,37].

### Implications for Anxiety

Of the 8 studies, 4 reported effects on anxiety. Arean et al [16], Dahne et al [33], Stiles-Shields et al [34], and Lüdtke et al [36] reported no results for anxiety. Only 50% (4/8) of the studies reported the desired outcome, and the results are inconsistent. Therefore, a clear implication of smartphone-based CBT cannot be drawn.

Two studies found no significant differences in the reduction of anxiety between the IG and CG. Ly et al [37] tested the reduction of anxiety by conducting an analysis of variance, which revealed a nonsignificant *F*_1,162.05_ value of 0.34 (P=.56). In addition, the effect sizes were particularly low, estimating Cohen *d* to be 0.03 (95% CI −2.30-2.37). Similar results were found in the study by Bakker et al [39] who used the Generalized Anxiety Disorder Scale (7-item). Although all groups reported a decrease in anxiety, the comparison of the 3 interventions revealed no significant values in the analysis of variance, and the eta-squared effect size was similarly low, ranging from 0.009 to 0.017.

The other 2 studies that reported anxiety as outcomes estimated a significant difference between the groups. Hur et al [38] used the State-Trait Anxiety Inventory-X2 tool and reported a Z value of −2.10 at follow-up in a nonparametric Wilcoxon rank-sum test. This was significant with a P value of .035. Both groups showed significantly decreased anxiety symptoms when baseline assessment and follow-up were compared (IG: Z=−2.91; P=.004; CG: Z=−2.51; P=.01). Another study, which reported significant effects of smartphone-based CBT on the reduction of anxiety, found that both IGs significantly declined anxiety relative to the waitlist CG. The *t* test for IG 1 estimated a t_236_ ratio of −2.48, which was highly significant (P=.01). IG 2, using a general version focused on self-esteem and acceptance app, expected a t_236_ ratio of −4.10, which was also highly significant (P<.001). Alternatively, no significant differences were found between both IGs. The estimated difference between the IGs was 1.06, favoring the general version of the intervention (t_237_=0.82; P=.41). Furthermore, IG 2 revealed a large effect size (Cohen *d* 0.92), whereas IG 1 showed only a small effect size (Cohen *d* 0.43), relative to the waitlist CG [35].

### Implications for Self-Efficacy or Self-Esteem

A total of 50% (4/8) of the included studies assessed the effects on self-efficacy or self-esteem. It was evident that different studies had underlying concepts and definitions of the term self-efficacy and self-esteem. Hur et al [38] and Lüdtke et al [36] measured self-esteem using the RSES, which assesses positive and negative feelings about the self as levels of self-esteem. The other 2 studies used various instruments to determine the associated outcomes of self-efficacy or self-esteem. Bakker et al [39] focused on coping abilities connected to self-efficacy and used the Coping Self-Efficacy Scale. However, Roepke et al [35] focused more on general self-efficacy, measuring how much people believe in achieving their goals despite difficulties. Therefore, the New General Self-Efficacy Scale was used.

Studies that used the RSES found no significant increase in self-esteem between the IG and CG. In a nonparametric Wilcoxon rank-sum test, Hur et al [38] estimated a Z value of −0.75, which was not significant (P=.45). Similar to this study, Lüdtke et al [36] conducted an *F* test, which was not significant as well (*F*_1,71_=1.464; P=.23).

The other 2 studies found significant increases in IG relative to the CG. IG 2 and IG 3 significantly increased self-efficacy (IG 2: *F*=4.86; P<.05; IG 3: *F*=14.95, P<.001). Furthermore, in mediation analyses, self-efficacy was found to be a significant mediator influencing anxiety, well-being, and depressive symptoms (P<.05) [39]. This was also evident in the study by Roepke et al [35]. IG 1 and IG 2 significantly increased self-efficacy relative to the CG at posttest. IG 2, which specifically focused on self-esteem, did not show higher increases in self-esteem relative to IG 1.

To conclude the implications for self-efficacy and self-esteem, inconsistent results were found. Due to the small number of studies and different assessment instruments, the effects of smartphone-based therapy on self-efficacy and self-esteem cannot be clearly shown. Different concepts of self-efficacy and self-esteem are present in the analyzed studies, which may have influenced the results. Furthermore, 1 study showed that self-efficacy was a significant mediator for depression symptoms, anxiety, and well-being.

### Implications for Quality of Life

Three studies reported that outcomes for quality of life were increased by smartphone-based CBT. In all studies, it was unclear if quality of life was measured in relation to health, as the studies used the term *Quality of life* instead of *health-related quality of life*. Due to the limited number of studies, it was not possible to draw implications that support the hypothesis of an increase in quality of life by applying smartphone-based CBT.

All 3 studies that directly measured quality of life could not establish significant effects between the IG and CG. Hur et al [38] reported a Z value of 1.19, which was not significant (P=.23). In addition, the analysis of variance of Ly et al [37] was also not significant (*F*_1,165.17_=1.06; P=.31). This was supported by the results of Lüdtke et al [36], who also did not find significant difference between the groups when analyzing the variance (*F*_1,70_=0.041; P=.84).

Concepts and definitions of quality of life are quite diverse. Therefore, there were 2 additional studies that reported subareas of quality of life. For example, Bakker et al [39] used the term *well-being* to describe quality of life. The authors showed that there was a significant increase in well-being in patients with depression that used IG 2 and IG 3 relative to the CG (IG 2: *F*=11.0, P<.001; IG 3: *F*=9.47, P<.01). Beyond this, Roepke et al [35] used the term life satisfaction and reported a significant increase in IG 1 and IG 2 (IG 1: t_236_=3.55, P<.001; IG 2: t_236_=2.71, P=.01). However, no significant difference was found between the 2 IGs.

Concluding the results of the studies, there are no clear implications concerning quality of life. Three studies that measured quality of life with established instruments [36] found no effects of smartphone-based therapy. Other studies that had a broader definition of life satisfaction or well-being could report effects. However, these aspects only used subareas of the concept of quality of life.

## Discussion

### Principal Findings

In conclusion, the comparability of the studies is limited due to various factors. This makes it difficult to interpret the obtained results. Due to various approaches to smartphone-based CBT, results differed between the studies. Significant differences were found in gender distributions between the groups, which might have biased the results. In addition, a lack of standardized presentation of the investigated endpoints made it difficult to generate clear implications. Although depression symptoms were mostly assessed with PHQ-9 or BDI-II, other investigated endpoints were assessed with different instruments, which made the comparability difficult. This may be due to the different objectives of the studies. For consistently reported endpoints, such as depression symptoms, the obtained results contradicted each other. Accordingly, no evident results could be found for smartphone-based CBT among patients with depression.

### Limitations

This systematic review has methodological limitations. This concerns the development of the search syntax. For its development, keywords and MeSH terms were used to identify suitable studies. The keywords of the studies were integrated into the search syntax for a larger number of hits. A complete coverage of all relevant keywords could not be guaranteed, which may have led to a bias in the study selection. The strength of this review was the systematic approach and the predefined procedure. To make the search procedure more comprehensible, the search syntax is presented in Multimedia Appendix 1.

Further limitations exist in the selection of studies. Studies were selected from the PubMed and Psyndex databases. Other fee-based databases were not considered and may have led to a selection bias. In addition, the 2 databases can be seen as the most relevant databases for the underlying research question. Furthermore, a selection bias can exist because of language. Only German and English studies were selected.

In total, 8 studies were included in the systematic review. The small number of studies made it difficult to prepare a funnel plot. Therefore, no information can be provided on possible publication biases. In addition, the included studies reported small effect sizes, which do not lead to a large dispersion around the no-effect line.

Finally, the use of the quality assessment tool must be reviewed critically. Owing to the subjective assignment of points to study design and performance, distortion cannot be excluded. To control this, quality assessment was discussed between the 2 reviewers. However, it can be stated that the applied instrument is reliable, as it has already been applied in several telemedical studies. This also accounts for the risk of bias assessment. For orientation, the Cochrane Handbook for Systematic Reviews of Intervention (Version 5.1.0) was used. Imprecise reporting of the included studies may have led to a different assessment of the studies. To control for this judgment, the quality assessment instrument and risk of bias tool were conducted multiple times, and the results were discussed between the 2 reviewers. The vague reporting of the authors was labeled as unclear risk of bias, as shown in Figure 3. However, comparing the quality assessment tool with the risk of bias tool, a concordance can be seen regarding the results.

The small number of included studies can be explained by the selection of study designs. Only RCTs were included to guarantee the highest evidence level. Therefore, this underlying work is a systematic review with a high evidence level. Furthermore, the included studies could report a high quality with a mean score of 11.43 points (median 12 points), indicating that the study design and performance of the RCTs were sufficient.

The content of this review was prepared by a qualitative synthesis of the study results. No statistical analysis of the individual studies was performed with the aid of a forest plot to evaluate the results. As a result, no meta-analysis was performed, which weakens the explanatory power of the results. The results of the review are based on the statistically determined values of the included studies, which were descriptively reported. A high heterogeneity between the studies was the leading argument for not performing a meta-analysis. Although the clinical heterogeneity was medium, the methodological heterogeneity was high. The included studies used different statistical tests to determine the effectiveness of smartphone-based CBTs. Due to the small number of included studies, a subgroup analysis was not considered to be useful. The heterogeneity of studies can further be explained by the research topic. Smartphone-based therapy is a relatively new research topic; therefore, current evidence is limited. CBT, as a profound treatment strategy, has experienced alterations and divisions into smaller treatment sections (eg, mindfulness-based therapy as a component of CBT). These alterations lead to an imprecise use of the definition of CBT. To control this, a sharper inclusion criterion in the beginning might have helped limit clinical and methodological heterogeneity. However, the observed heterogeneity between the studies is not unique to this work because Firth et al [39] determined similar problems with heterogeneity when evaluating the effectiveness of smartphone-based interventions. As children and minorities were excluded, the obtained results were not conclusive for these groups.

### Comparison With Prior Work

To place the obtained results in the scientific context, a systematic review and meta-analysis of Firth et al [42] was identified, which showed a significant reduction in depressive symptoms when smartphone-based mental health interventions were applied. In agreement with the results of this review, the authors of the study only found small to medium effect sizes of the intervention. These effect sizes differed when inactive or active CGs were used for comparison. These results cannot be supported by this review, but the statement is limited because a meta-analysis was not conducted. Furthermore, this review had a more detailed approach to mental health interventions. The underlying review assessed the effectiveness of a smartphone-based CBT. However, Firth et al [42] chose a broader approach and included all studies that were classified as a mental health intervention with no scope on specific treatment strategies. This might be a reason why the authors could include 18 studies, whereas this review only found 8 eligible studies. Many studies that were included by Firth et al [42] were excluded in this review for a comprehensible reason. The inclusion of the same studies showed that this review identified the most relevant studies in the research area. Multiple studies that were performed after 2017 were not included by Firth et al [42]. Therefore, this review can be viewed as an update of the effectiveness of smartphone-based therapy among patients with depression. It should also be noted that this review particularly assessed smartphone-based CBT approaches. Another review by Bakker et al [43] formulated evidence-based recommendations, which stated that CBT is a well-researched therapeutic technique for depression. In particular, the effectiveness of computerized CBT was proven by 2 meta-analyses [44,45]. However, recommendations that smartphone-based CBT is effective cannot be supported by this review. The results of this review are inconclusive for the observed endpoints of depression, anxiety, self-efficacy or self-esteem, and quality of life.

### Conclusions

It is apparent that evidence for smartphone-based CBT is limited. Although the economic and public health importance of depression is often mentioned, there is a lack of RCTs with good quality. The effects of smartphone-based apps are insufficiently analyzed. Therefore, there is need for further research. Current research efforts are more focused on determining the feasibility and moderators that are present when applying smartphone-based CBT. These studies fail to determine the effectiveness of technologies that are needed to build the basis for discussions about integrating new treatment methods in reimbursement systems.

The lack of high-quality studies can also be associated with whether effectiveness can only be determined by conducting RCTs. Recently, there has been a rising discussion about alternative study designs that are more flexible than rigor RCT and therefore more suitable for evaluating mHealth technologies [46]. mHealth and other health technologies are more difficult to evaluate than pharmaceutical products. The task of science is to develop new methods and assessment tools that consistently represent the effects of smartphone-based treatments. Efforts toward a standardized reporting of results in the sense of evidence-based medicine would facilitate the comparability and the implementation of systematic reviews and meta-analyses. Actions such as the development of consolidated standards of reporting trials of electronic and mHealth apps and web-based telehealth (CONSORT-EHEALTH) are appreciated and should be recommended for researchers who conduct clinical trials using mHealth technologies [47]. This would facilitate and simplify the execution of systematic reviews to determine evidence-based recommendations for digital health.

For clinical practice, it is crucial to answer the question of how smartphone-based treatment approaches can be integrated into the current routines. Due to legal limitations (eg, ban of remote treatment in Germany), it is clear that these new treatment approaches should add current care and not replace it. A good combination of remote treatment and face-to-face consultation can be considered the gold standard.

Smartphone-based therapy must not interfere with everyday practice or be perceived as disturbing them. Here, technology developers are called upon to develop such a technology for practice. Further training courses for practitioners are of particular importance with regard to the acceptance of smartphone-based therapy.

mHealth interventions often face high attrition rates, which hinders the practical use of those technologies. Therefore, it is of major importance that mHealth interventions are designed with a participatory approach. By integrating users, practitioners, and developers, more effective interventions can be created that are actually used in clinical practice. Concepts such as human-centered design in health technology development should be prioritized to provide digital public health interventions and help patients with a high burden of disease [48,49]. As mHealth interventions rely on user interaction and application, it is important that potential users understand the concepts and applications of smartphone-based therapy. Information and exercises (eg, mood tracking or cognitive behavioral tasks) that are not fully understood by its users lead to attrition. Practitioners or caregivers are seen as being in a position to provide training and introduction and to support the patient in the process of use. In the beginning, this might be a hindering factor for the practitioner to use smartphone-based therapy, but the benefits could be long lasting and establish a shared-decision partnership between the practitioner and the patient. It should be noted that digital competence is always linked to literacy and a certain degree of education. To provide smartphone-based therapy to all population groups, a special focus should be placed on minorities and population groups that face socioeconomical disadvantages. Those population groups need major support and training to ensure that they profit from mHealth interventions.

Fields of action for policy are seen in the development of mHealth structures. In this context, the *Global* *Strategy for Digital Health*, published by the WHO in 2019, can be acclaimed, as their aim is to apply digital health by a vision of health for all. More precisely, the WHO emphasizes that digital health adoption is a decision of the respective country, which requires a unified strategy that integrates leadership, financial, organizational, human, and technological resources. In addition, the use of digital technologies is needed to support equity in terms of access to care by being people-centered, evidence-based, and ethically appropriate [50]. As mHealth competences and socioeconomic determinants are not equally distributed, people with a low socioeconomic status and low competencies need to be supported more. Therefore, policy makers should especially consider those aspects when creating or passing new laws to establish mHealth structures. It is of major importance that digital innovations do not widen the gap of social disparity, which subsequently results in health inequity.

Transparency is an important aspect that empowers patients to make their own decisions [51]. Especially in smartphone-based therapy, it can be seen that the market of apps (eg, Appstore or Android Store) is highly unregulated and policy makers are required to develop regulatory instruments to ensure patients’ safety. By regulating the market, technological and scientific innovation can be slowed down. However, it is mandatory that users are able to fully understand whether the app is a medical device or an unregulated app with no evidence-based background [52,53]. Furthermore, the development and functioning of apps is often compared with a blackbox because data use and underlying mechanisms are often hidden by the developers. This hinders transparency, as users should fully understand and know what happens with their data. Open-source projects can be a solution to empower users to understand the mechanisms of mHealth. Through strong involvement of organized interests in health care systems, policy makers must ensure that all involved actors in ensuring care are committed to adhere to the framework conditions. The General Data Protection Regulation of the European Union that increases data protection must also be considered in mHealth interventions [54].

Other areas of responsibility, which need to be addressed by policy makers, include financing issues of mHealth. Although benefits of certain forms of mHealth apps have been proven, the widespread implementation of the technology is failing due to financing problems. Financing of innovations as pilot studies is not efficient in the long term. Accordingly, after successful pilot studies, large-scale studies are needed, and the transfer to standard care should be achieved without delay. The obligation of policy makers is to sustainably fund these efforts to modernize and digitalize health care [55].

## Appendix

Multimedia Appendix 1 Search Syntax.

Multimedia Appendix 2 Patient characteristics.

Multimedia Appendix 3 Study results.

Multimedia Appendix 4 Quality assessment of the included studies.

## Abbreviations

BDI-II: Beck Depression Inventory-II
CBT: cognitive behavioral therapy
CG: control group
IG: intervention group
MeSH: Medical Subject Headings
mHealth: mobile health
PHQ: Patient Health Questionnaire
RCT: randomized controlled trial
RSES: Rosenberg Self-Esteem Scale
WHO: World Health Organization
