# Supplementary material for: Effectiveness of Smartphone-Based Cognitive Behavioral Therapy Among Patients With Major Depression: Systematic Review of Health Implications
Source: JMIR Mhealth Uhealth. 2021 Feb 10;9(2):e24703. doi: 10.2196/24703 (PMC7904402; doi:10.2196/24703)
Supplement: Multimedia Appendix 1 [file mhealth_v9i2e24703_app1.docx]

Appendix 1 – Search Syntax

**Databases:** Pubmed and Psyndex

Date of search: May 2020

1. Depression (MESH) OR depress* OR major depression OR depressive disorder (MESH) OR depressive disorder, major (MESH) OR depressive episode OR unipolar depression
2. Cognitive therapy OR behavior therapy (MESH) OR cognitive behavioral therapy (MESH) OR acceptance and commitment therapy (MESH) OR mindfulness
3. Smartphone (MESH) OR computers, handheld (MESH) OR mHealth OR mobile health OR smartphone-delivered therapy OR smartphone-based therapy OR internet-based interventions (MESH) OR mobile applications (MESH)
4. # 1 AND # 2 AND # 3

**Filters:** published in the last 5 years

Randomized Controlled Trials
