# Supplementary material for: Effectiveness of Smartphone-Based Cognitive Behavioral Therapy Among Patients With Major Depression: Systematic Review of Health Implications
Source: JMIR Mhealth Uhealth. 2021 Feb 10;9(2):e24703. doi: 10.2196/24703 (PMC7904402; doi:10.2196/24703)
Supplement: Multimedia Appendix 2 [file mhealth_v9i2e24703_app2.docx]

Appendix 2 – patient characteristics

| Study | Recruitment | Mean age (M± SD) | | Gender distribution (abs. number and percent) n (%) | | Baseline depression symptoms (M ± SD) | | | |
| --- | --- | --- | --- | --- | --- | --- | --- | --- | --- |
|  |  |  |  |  |  | PHQ-9 | | BDI-II | |
|  |  | IG | CG | IG | CG | IG | CG | IG | CG |
| Roepke et al. 2015 [35] | General Population | IG 1:  42.28 (12.56)  IG 2:  37.99 (11.31) | 40.27 (13.06) | IG 1:  36 (38.71) m  57 (61.29) f  IG 2:  25 (25.77) m  72 (74.23) f | 22 (23.66) m  71 (76.34) f | NA | NA | NA | NA |
| Ly et al. 2015 [37] | General Population | 30.2 (11.9) | 31.0 (11.0) | 16 (34.8) m  30 (65.2) f | 12 (25.5) m  35 (74.5) f | 15.39 (4.73) | 15.30 (4.49) | 28.96 (8.07) | 27.32 (7.89) |
| Arean et al. 2016 [16] | General Population | IG 1:  34.9 (12.3)  IG 2:  33.4 (10.9) | 33.6 (12.3) | IG 1:  51 (24.2) m  158 (75.8) f  IG 2:  48 (23) m  163 (77) f | 33 (16) m  173 (84) f | IG 1:  13.76 (4.9)  IG 2:  13.51 (5.1) | 13.64 (4.9) | NA | NA |
| Bakker et al. 2018 [39] | General Population | IG 1:  36.1 (11.5)  IG 2:  33.3 (12.8)  IG 3:  33.8 (13.6) | 33.6 (10.7) | IG 1:  4 (7) m  52 (93) f  IG 2:  9 (18) m  41 (82) f  IG 3:  12 (21) m  44 (79) f | 14 (22) m  50 (78) f | IG 1:  8.04 (4.9)  IG 2:  10.94 (6.22)  IG 3:  11.25 (6.54) | 8.55 (6.62) | NA | NA |
| Hur et al. 2018 [38] | General population and outpatient clinics | 24.76 (3.70) | 22.65 (2.42) | 2 (11.76) m  15 (88.24) f | 2 (11.76) m  15 (88.24) f | NA | NA | 22.65 (7.94) | 25.59 (7.93) |
| Lüdtke et al. 2018 [36] | General population and outpatient clinics | 41.20 (11.86) | 44.57 (10.69) | 8 (18.18) m  36 (81.82) f | 11 (25) m  33 (75) f | 11.61 (6.14) | 12.77 (6.40) | NA | NA |
| Dahne et al. 2019 [33] | Outpatient clinics | 44.67 (13.95) | CG 1:  43.00 (13.63)  CG 2:  43.11 (11.88) | 4 (16.7) m  20 (83.3) f | CG 1:  4 (21.1) m  15 (78.9) f  CG 2:  1 (15.4) m  8 (84.6) f | NA | NA | 28.08 (7.83) | CG 1:  28.63 (11.3)  CG 2:  32.33 (13.03) |
| Stiles-Shields et al. 2019 [34] | General Population | IG 1:  35.5 (17.2)  IG 2:  43.1 (11.5) | 34.1 (11.0) | IG 1:  1 (10) m  9 (90) f  IG 2:  4 (40) m  6 (60) f | 2 (20) m  8 (80) f | IG 1:  15.20 (5.49)  IG 2:  17.00 (4.62) | 16.10 (3.76) | NA | NA |

Source: own representation; IG: intervention group; CG: control group; NA: not specified; m: male; f: female
