# Supplementary material for: Effectiveness of Smartphone-Based Cognitive Behavioral Therapy Among Patients With Major Depression: Systematic Review of Health Implications
Source: JMIR Mhealth Uhealth. 2021 Feb 10;9(2):e24703. doi: 10.2196/24703 (PMC7904402; doi:10.2196/24703)
Supplement: Multimedia Appendix 3 [file mhealth_v9i2e24703_app3.docx]

Multimedia Appendix 3. Study results.

| Study | Depressive symptoms | anxiety | Self-efficacy/self-esteem | Quality of life |
| --- | --- | --- | --- | --- |
| Roepke et al. 2015 [35] | Significant decrease of CES-D in IG 1 compared to CG at posttest (t_237_= -2.80; *P*<.01)  Significant decrease between IG 2 and CG at posttest (t_237_= -3.73; *P*< 0.001)  No significant difference between IG 1 and IG 2 (t_237_= 0.82; *P*=.41) | Significant decrease of GAD-7 in IG 1 compared to CG at posttest (t_236_= -2.48; *P*<.01)  Significant difference between IG 2 and CG at posttest (t_236_= -4.10; *P*<.001)  No significant difference between IG 1 and IG 2 (t_237_= 0.82; *P*=.41)  Greater effect sizes for IG 2 than for IG 1 (Cohen’s d: IG1= 0.43; IG2= 0.92) | Significant increase of self-efficacy in IG 1 compared to CG at posttest (t_234_= 3.59; *P* <.001)  Significant difference between IG 2 and CG at posttest (t_234_= 2.55; *P*=.01)  No significant difference between IG 1 and IG 2 (values unspecified) | Significant increase of life satisfaction in IG 1 compared to CG at posttest (t_236_= 3.55; *P*<.001)  Significant difference between IG 2 and CG at posttest (t_236_= 2.71; *P*=.01)  No significant difference between IG 1 and IG 2 (values unspecified) |
| Ly et al. 2015 [37] | No significant difference of BDI-II scores between IG and CG at follow-up (F_1, 171.81_= 0.13; *P*=.72) and PHQ-9 (F_1, 911.85_= 0.11; *P*=.74) | No significant differences between IG and CG at follow-up (F_1, 162.05_= 0.34; *P*=.56) | NA | No significant differences between IG and CG at follow-up (F_1, 165.17_= 1.06; *P*=.31) |
| Arean et al. 2016 [16] | No significant difference between IG 1 and IG 2 compared to CG (B= -0.01; *P*=.90)  No significant difference between IG and CG for mildly depressed subgroup  Significant decrease of PHQ-9 in IG 2 compared to CG at week 12 (t_201_= -2.36, *P*=.02) | NA | NA | NA |
| Bakker et al. 2018 [39] | Significant decrease of PHQ-9 in IG 2 compared to CG (F= 4.39; *P*<.05) and in IG 3 compared to CG (F= 4.24; *P*<.05) | No significant decrease between IG (IG1-3) and CG (*P*<.05) | Significant increase of self-efficacy in IG 2 compared to CG (F= 4.86; *P*<.05) and in IG 3 compared to CG (F= 14.95, *P*<.001)  Significant overall mediation role for IG regarding anxiety, well-being and depressive symptoms (*P*<.05) | Significant increase of well-being in IG 2 compared to CG (F= 11.0; *P*<.001) and in IG 3 compared to CG (F= 9.47, *P*<.01) |
| Hur et al. 2018 [38] | No significant difference between IG and CG at follow-up (Z= -1.90; *P*=.06) | Significant decrease of STAI-X2 in IG compared to CG at follow-up (Z= -2.10; *P*=.04) | No significant difference between IG and CG at follow-up (Z= -0.75; *P*=.45) | No significant differences between IG and CG (Z= -1.19; *P*=.23) |
| Lüdtke et al. 2018 [36] | No significant difference of PHQ-9 between IG and CG (F_1, 71_= 0.173; *P*=.68) | NA | No significant difference between IG and CG (F_1, 71_= 1.464; *P*=.23) | No significant difference between IG and CG (F_1, 70_= 0.041; *P*=.84) |
| Dahne et al. 2019 [33] | No significant difference between IG 1 and CG2 (mean difference: -3.94; *P*=.18), IG 1 compared to CG1 (mean difference: 1.74; *P*=.55), CG1 compared to CG2 (mean difference: -5.69; *P*=.07) | NA | NA | NA |
| Stiles-Shields et al. 2019 [34] | Significant decrease of PHQ-9 in IG compared to CG (F_6, 72_= 2.78; *P*=.02)  Significant decrease of PHQ-9 in IG 2 compared to CG (*P*=.03)  No significant difference between IG 1 and IG 2 and CG (*P*>.2) | NA | NA | NA |

Source: own representation; IG: intervention group; CG: control group; NA: not specified; PHQ: Patient Health Questionnaire; BDI: Beck Depression Inventory; CES-D: Center for Epidemiologic Studies Depression Scale
