# Supplementary material for: Effectiveness of Smartphone-Based Cognitive Behavioral Therapy Among Patients With Major Depression: Systematic Review of Health Implications
Source: JMIR Mhealth Uhealth. 2021 Feb 10;9(2):e24703. doi: 10.2196/24703 (PMC7904402; doi:10.2196/24703)
Supplement: Multimedia Appendix 4 [file mhealth_v9i2e24703_app4.docx]

Appendix 4 – Quality assessment

| **category study** | **Arean et al. 2016** | **Bakker et al. 2018** | **Dahne et al. 2019** | **Hur et al. 2018** |
| --- | --- | --- | --- | --- |
| **Study design:** | | | | |
| 1. Big RCT   (≥ 50 participants each intervention arm): 5 points | 5 | 5 | - | - |
| 1. small RCT   (< 50 participants each intervention arm): 3 points | - | - | 3 | 3 |
| 1. prospective non-randomized trial: 2 points | - | - | - | - |
| 1. retrospective non-randomized trial: 1 point | - | - | - | - |
| If RCT:   1. Randomization appropriately described? 2. Blinded? 3. Blinding appropriately described?   (half a point is subtracted if the information is missing) | 1. Yes 2. No (-0.5) 3. No (-0.5) | 1. Yes 2. Yes 3. No (-0.5) | 1. No (-0.5) 2. No (-0.5) 3. No (-0.5) | 1. Yes 2. No (-0.5) 3. No (-0.5) |
| **Study performance:**  Score (0= no information, 1= information limited, 2= information satisfactory) | | | | |
| 1. Patient selection | 2 | 1 | 2 | 2 |
| 1. Description of the intervention | 2 | 2 | 2 | 2 |
| 1. Specification and analysis of study (intention-to-treat) | 2 | 2 | 1 | 1 |
| 1. Patient disposal | 2 | 2 | 1 | 1 |
| 1. Outcomes reported | 1 | 2 | 1 | 1 |
| **Overall score** | **13** | **13,5** | **8,5** | **9** |
| **Quality category** | **A** | **A** | **C** | **C** |

| **category study** | **Ly et al. 2015** | **Lüdtke et al. 2018** | **Roepke et al. 2015** | **Stiles-Shields et al. 2019** |
| --- | --- | --- | --- | --- |
| **Study design:** | | | | |
| 1. Big RCT   (≥ 50 participants each intervention arm): 5 points | - | - | 5 | - |
| 1. small RCT   (< 50 participants each intervention arm): 3 points | 3 | 3 | - | 3 |
| 1. prospective non-randomized trial: 2 points | - | - | - | - |
| 1. retrospective non-randomized trial: 1 point | - | - | - | - |
| If RCT:   1. Randomization appropriately described? 2. Blinded? 3. Blinding appropriately described?   (half a point is subtracted if the information is missing) | 1. Yes 2. Yes 3. No (-0.5) | 1. No (-0.5) 2. No (-0.5) 3. No (-0.5) | 1. Yes 2. No (-0.5) 3. No (-0.5) | 1. Yes 2. Yes 3. No (-0.5) |
| **Study performance:**  Score (0= no information, 1= information limited, 2= information satisfactory) | | | | |
| 1. Patient selection | 2 | 2 | 2 | 2 |
| 1. Description of the intervention | 2 | 2 | 2 | 2 |
| 1. Specification and analysis of study (intention-to-treat) | 2 | 2 | 2 | 1 |
| 1. Patient disposal | 2 | 2 | 2 | 1 |
| 1. Outcomes reported | 2 | 2 | 2 | 1 |
| **Overall score** | **12,5** | **11,5** | **14** | **9,5** |
| **Quality category** | **A** | **A** | **A** | **B** |
| 11,5 – 15,0 points High quality (A) 5,5 – 7,0 points Poor to fair quality (D)  9,5 – 11,0 points Good quality (B) 1,0 – 5,0 points Poor quality (E)  7,5 – 9,0 points Fair to good quality (C) | | | | |
